# DNA Methylation and Demethylation Are Regulated by Functional DNA Methyltransferases and DnTET Enzymes in *Diuraphis noxia*

**DOI:** 10.3389/fgene.2020.00452

**Published:** 2020-06-23

**Authors:** Pieter H. du Preez, Kelly Breeds, N. Francois V. Burger, Hendrik W. Swiegers, J. Christoff Truter, Anna-Maria Botha

**Affiliations:** Department of Genetics, Stellenbosch University, Stellenbosch, South Africa

**Keywords:** whole genome bisulfite sequencing, DNMT and TET, global methylation (5mC), global hydroxymethylation (5hmC), Russian wheat aphid

## Abstract

Aphids are economically important insect pests of crops worldwide. Despite resistant varieties being available, resistance is continuously challenged and eventually broken down, posing a threat to food security. In the current study, the epigenome of two related Russian wheat aphid (*Diuraphis noxia*, Kurdjumov) biotypes (i.e., SA1 and SAM) that differ in virulence was investigated to elucidate its role in virulence in this species. Whole genome bisulfite sequencing covered a total of 6,846,597,083 cytosine bases for SA1 and 7,397,965,699 cytosine bases for SAM, respectively, of which a total of 70,861,462 bases (SA1) and 74, 073,939 bases (SAM) were methylated, representing 1.126 ± 0.321% (SA1) and 1.105 ± 0.295% (SAM) methylation in their genomes. The sequence reads were analyzed for contexts of DNA methylation and the results revealed that RWA has methylation in all contexts (CpG, CHG and CHH), with the majority of methylation within the CpG context (± 5.19%), while the other contexts show much lower levels of methylation (CHG − ± 0.27%; CHH − ± 0.34%). The top strand was slightly (0.02%) more methylated than the bottom strand. Of the 35,493 genes that mapped, we also analyzed the contexts of methylation of each of these and found that the CpG methylation was much higher in genic regions than in intergenic regions. The CHG and CHH levels did not differ between genic and intergenic regions. The exonic regions of genes were more methylated (±0.56%) than the intronic regions. We also measured the 5mC and 5hmC levels between the aphid biotypes, and found little difference in 5mC levels between the biotypes, but much higher levels of 5hmC in the virulent SAM. RWA had two homologs of each of the *DNA methyltransferases 1* (*DNMT1a* and *DNMT1b*) and *DNMT3s* (*DNMT3a* and *DNMT3b*), but only a single *DNMT2*, with only the expression of *DNMT3* that differed significantly between the two RWA biotypes. RWA has a single ortholog of Ten eleven translocase (*DnTET*) in the genome. Feeding studies show that the more virulent RWA biotype SAM upregulate *DnDNMT3* and *DnTET* in response to wheat expressing antibiosis and antixenosis.

## Introduction

*Diuraphis noxia* (Kurdjumov, Hemiptera: Aphididae—or Russian wheat aphid, RWA) biotypes are morphologically similar, yet display vast differences in their capacity to damage wheat cultivars upon feeding (i.e., their virulence) (Botha, 2013). In South Africa, the virulence of the four wild type and the mutant RWA biotypes is as follows in order from least to most virulent: SA1 < SA2 < SA3 < SA4 < SAM (Swanevelder et al., 2010; Jankielsohn, 2016). Despite the emergence of new RWA biotypes in South Africa (Tolmay et al., 2007; Jankielsohn, 2011, 2016), and other parts of the world, including the United States of America (USA) (Haley et al., 2004; Burd et al., 2006; Randolph et al., 2009) and Argentina (Clua et al., 2004), the molecular mechanism(s) underlying the development of new biotypes is currently unknown (Shufran and Payton, 2009; Botha et al., 2014a). The known genealogy of SA1 and SAM (Swanevelder et al., 2010), their genetic similarity (Burger and Botha, 2017) and their position on either end of the virulence spectrum, renders them particularly useful in the present study, to improve the understanding of the process of biotypification. The possibility of a link between RWA methylation and biotype virulence has previously been suggested (Gong et al., 2012; Breeds et al., 2018). In 2012, Gong et al. investigated the methylation of four genes encoding salivary gland proteins (putative effector genes) in RWA biotypes US1 and US2, and found these genes to be differentially methylated in the different biotypes. In the initial investigation of South African RWA methylation (Breeds et al., 2018), the different biotypes exhibited different banding patterns (after restriction of their DNA with methylation-sensitive enzymes), methylation levels and methylation trends, all of which support a role for methylation in biotypification.

The epigenetic modification of DNA methylation involves the covalent addition of a methyl group to the 5′ position of cytosine (Glastad et al., 2011; Lyko and Maleszka, 2011). In insects, methylation occurs predominantly within genes (Walsh et al., 2010; Zemach et al., 2010; Glastad et al., 2011; Lyko and Maleszka, 2011), where to date it is reported to perform two major functions. Firstly, intragenic methylation affects alternative splicing by recruiting or interfering with different DNA binding factors (Hunt et al., 2013b; Glastad et al., 2014; Yan et al., 2015), and secondly, it prevents the initiation of spurious transcription at cryptic binding sites within genes (Hunt et al., 2010, 2013a,b). Three classes of DNA methyltransferase (DNMT) proteins are involved in methylation of DNA and these perform different functions, with DNMT3 and DNMT1 establishing and maintaining methylation patterns, respectively, but with a less clear function for DNMT2. This class is known to show strong conservation in sequence and is suggested to be an ancient DNA methyltransferase that changed its substrate specificity from DNA to tRNA (Sunita et al., 2008; Iyer et al., 2011; Jurkowski and Jeltsch, 2011; Raddatz et al., 2013). Insects have a variety of combinations of the *DNMT* genes, with some lineages having lost one (e.g., *Bombyx mori* and *Triboleum castaneum*) or two (e.g., *Drosophila melanogaster* and *Anopheles gambiae*) classes of *DNMTs*, and others having multiple homologs (e.g., *Apis mellifera, Nasonia vitripennis*, and *Acyrthosiphon pisum*) within a certain *DNMT* class (Kunert et al., 2003; Marhold et al., 2004; Walsh et al., 2010; Xiang et al., 2010; Glastad et al., 2011; Feliciello et al., 2013). Despite their important function in DNA methylation, knowledge of RWA *DNMTs* is still lacking.

DNA methylation is removed through the process of demethylation, which can occur both passively and actively, with 5-hydroxymethylcytosine (5hmC) being a measurable intermediate of one of the active demethylation pathways (Branco et al., 2012; Kohli and Zhang, 2013). Hydroxymethylcytosine is formed through the oxidation of 5mC by ten-eleven translocation enzymes (TETs) (Tahiliani et al., 2009; Shen et al., 2014). The presence of 5hmC has only been reported in a few insects including *A. mellifera, T. castaneum, N. vitripennis* and *D. melanogaster* (Cingolani et al., 2013; Feliciello et al., 2013; Wojciechowski et al., 2014; Delatte et al., 2016; Pegoraro et al., 2016; Rasmussen et al., 2016). To determine the presence and extent of 5hmC in the RWA, an antibody specific to 5hmC was used, providing the first insight into RWA demethylation.

The objective in this study was firstly to sequence and compare the epigenome of RWA biotypes SA1 and SAM, and determine the level, location (e.g., intergenic or genic, exonic or intronic), and contexts of DNA methylation (i.e., CpG, CHH, CHG) within the genomes of these RWA biotypes with differential virulence. Secondly, to quantify global methylation (5mC) and demethylation (5hmC) in the South African biotypes with shared genealogy; and thirdly, to characterize the DNA methyltransferases (*DNMTs*) and ten-eleven translocase enzyme-like (*TET*) genes and expression in these aphids, to relate these observations to the reported difference in virulence levels of the South African RWA biotypes SA1 and SAM.

## Materials and Methods

### Aphid Rearing

For whole genome bisulfite sequencing and measurement of global methylation (5mC) and hydroxymethylation (5hmC) levels, colonies of apterous parthenogenetic female aphids of South African RWA biotypes SA1 and SAM were separately established in BugDorm cages (MegaView Science Education Services Co. Ltd., Taiwan) in an insectary with the following conditions: 22.5 ± 2.5°C, 40% relative humidity, and continuous artificial lighting from high pressure sodium lamps as previously described (Breeds et al., 2018). In all instances triplicate colonies of each biotype were established. Stock colonies of RWA biotype SA1 were maintained on the wheat line Tugela (susceptible) and biotype SAM on the near isogenic wheat line Tugela-*Dn1* (resistant). To avoid any environmental effects due to feeding on different wheat plants, aphid biotypes were transferred to the susceptible cultivar “SST356” 1 month prior to DNA extraction for the whole genome bisulfite sequencing. In all instances, treatments were conducted using separate BugDorm cages in triplicate (*n* = 3 × 2).

For *DnDNMT* expression analysis (0h), RWA biotype SA1 was maintained on the “SST 356” wheat cultivar (susceptible), while the highly virulent SAM biotype was maintained on “SST 398” (RWA resistant), and then transferred to the susceptible “SST 356” wheat cultivar 1 month prior to RNA extraction and cDNA synthesis.

For the RNAseq analysis, colonies of apterous parthenogenetic female aphids of South African RWA biotypes SA1 and SAM were separately established in BugDorm cages (MegaView Science Education Services Co. Ltd., Taiwan) in an insectary with the following conditions: 22.5 ± 2.5°C, 40% relative humidity, and continuous artificial lighting from high pressure sodium lamps as previously described (Breeds et al., 2018). RWA biotype SA1 was maintained on the wheat line Tugela (susceptible) and biotype SAM on the near isogenic wheat line Tugela-*Dn5* (resistant). Multiple individual replicates, consisting of 50 aphids of various life stages, were collected for each biotype. Collected aphids were flash frozen in liquid nitrogen and RNA was extracted following the protocol of Qiagen's RNeasy RNA extraction kit performing the optional on column Qiagen DNase treatment. Extracted RNA was assessed for quality through both bleach-gel electrophoresis (Aranda et al., 2012) and with an Agilent 2100 Bioanalyser using the RNA Nano 6000 kit (Babu and Gassmann, 2016). Three RNA samples, from each biotype, representing three biological repeats, with the highest RIN values (at least above 7) were used in subsequent analysis.

For the *DnDNMT* and *DnTET* host-shifts, RWA biotypes SA1 and SAM were maintained on the susceptible “SST 356” wheat cultivar, and then transferred to either near isogenic wheat lines Tugela (susceptible), or Tugela-*Dn1* (resistant), or Tugela-*Dn5* (resistant) prior to RNA extraction and cDNA synthesis. Aphids were sampled at 0, 6, and 48 h post host-shifting. In all instances, treatments were conducted using separate BugDorm cages using separate plants in triplicate (*n* = 3 × 2).

For the quantitation of DNMT proteins, both biotypes were maintained on the “SST 356” wheat cultivar before protein extraction. In all instances, treatments were conducted using separate BugDorm cages in triplicate (*n* = 3 × 2). All SST cultivars were obtained from SENSAKO (Pty) Ltd., (South Africa).

### Identification, Cloning and Sequencing of RWA *DNMTs* and *Ten Eleven Translocation-Like (TET-Like) Genes*

DNMT and TET sequences of the pea aphid (*Acyrthosiphon pisum*) were obtained through GenBank and used as BLAST (Altschul et al., 1997) queries against the NCBI's non-redundant (nr) database to obtain homologs from the Class Insecta. The obtained sequences were then aligned using MAFFT v.7.4 (Katoh and Standley, 2013) and through use of maximum parsimony the obtained sequences were phylogenetically rendered through use of PAUP v4.0a136.

Primers were designed (Table S1) to amplify the transcripts of identified RWA *DNMTs* and *TET-like genes* using Primer3 (Rozen and Skaletsky, 2000). The primers were then used in a primer BLAST analysis against the RWA SAM biotype reference genome (GCA_001465515.1) to ensure they only matched genes of interest. RNA extractions and cDNA synthesis were performed for both RWA biotypes SA1 and SAM as previously described (Burger et al., 2017).

PCR reactions for sequencing were performed using Phusion High-Fidelity DNA Polymerase (NEB) and following the manufacturer's protocol. PCR products were then ligated into the pTZ57R/T vector (InsTAclone PCR cloning kit, Thermo Scientific) overnight at 4°C. For PCR reactions showing non-specific amplification, gel fragments containing bands of the expected product size were excised and subjected to five freeze-thaw cycles (liquid nitrogen/60°C oven) in 20 μl of distilled water and the obtained DNA was quantified through spectrophotometry (NanoDrop 2000, Thermo). Based on these results, differing amounts of freeze-thawed DNA were used, in accordance with the kit's recommendations on the optimal quantity of PCR product for ligation.

Transformation of DH5α competent cells (Thermo Scientific) was performed through heat shock following the manufacturers' protocol and recombinant colonies were cultured and screened as previously performed (Burger et al., 2017). Plasmid minipreps (derived from at least one colony per PCR product) were submitted to the Central Analytical Facility (CAF) of Stellenbosch University for bi-directional Sanger sequencing using universal M13 forward and reverse primers (Table S1).

After Sanger sequencing, raw sequences were imported into Geneious v.9.1.8 and trimmed on either end to remove poor quality or ambiguous base calls. A VecScreen BLAST (http://www.ncbi.nlm.nih.gov/tools/vecscreen/) was then performed using the trimmed sequences to remove any vector DNA. The sequences for both SA1 and SAM biotypes (at least one forward and one reverse per PCR product) were aligned with the respective gene from which primers were designed using Primer 3 (Sievers et al., 2011).

### Sequencing, Transcriptome Assembly, and Quality Control

RNA samples were sent for sequencing at Macrogen Inc., South Korea where six libraries were prepared using the TruSeq Stranded mRNA Sample Preparation Guide, Part #15031047 Rev. E. Paired-end library construction was performed using the Illumina TruSeq stranded mRNA kit and the subsequent libraries were sequenced on the Illumina NovaSeq 6000 system to obtain 100 bp paired-end reads for three replicates of both the *Diuraphis noxia* SAM and SA1 biotypes.

Raw reads obtained from the NovaSeq 6000 system were analyzed through use of FASTQC (Andrews, 2010) and trimmed of all poor quality reads and sequencing adaptors through use of Trimmomatic (Bolger et al., 2014). The trimmed reads were then used to perform a strand specific *de novo* assembly through use of the Trinity software suite (Haas et al., 2013). The assembled transcriptome's quality was assessed through mapping the reads back to the assembled transcripts using Bowtie2 (Langmead and Salzberg, 2012), and to assess the percentage of reads utilized to construct the transcriptome. A BUSCO v4.2 (Simão et al., 2015) analysis was also performed using the Insecta homolog set (accessed on 2020/02, https://buscos.ezlab.org/datasets/prerelease/viridiplantae_odb10.tar.gz) to establish the number of represented essential orthologs. To assess if sequencing depth was adequate to generate a high quality *de novo* assembly, successively increasing sub-samplings of total sequencing data for individual samples were performed and assembled separately. These were then compared through the use of BLASTx to the NCBI's nr (protein) database, and the SwissProt uniprotKB and TrEMBL databases to assess the number of full-length BLAST matches obtained for the assembled transcripts from the differently sized assemblies.

Basic statistics such as the number of transcripts, transcript average length, transcript average %GC content, transcript N50 and transcript Ex90N50 were also calculated. All transcripts were analyzed through use of OmicsBox v1.2 (OmicsBox—Bioinformatics Made Easy, BioBam Bioinformatics, March 3, 2019, https://www.biobam.com/omicsbox) by performing BLASTx and BLASTn searches, respectively to the NCBI's nr and nt databases (accessed on 2019/08/21). Blast2GO (Götz et al., 2008) was then used to assign gene ontologies (GO) and KOG terms to all transcripts.

To compare the differential gene expression between the least and most virulent biotypes, transcript abundance quantification was performed using RSEM (Li and Dewey, 2011) for each sample using the obtained *de novo* transcripts. Average expression and the coefficient of variation was calculated per gene for the two biotypes SA1 and SAM separately. For this purpose FPKM (fragments per kilobase of transcript per million) values were used but also estimated by RSEM. We also identified differentially expressed (DE) genes between biotypes SA1 and SAM using edgeR (Robinson et al., 2010) based on gene-level expected counts estimated by RSEM. Only genes with greater than two counts-per-million in at least three samples were retained for DE analysis and we considered genes DE if they had a fold-change (FC) ≥1.5 and *p* < 0.05 after adjusting for multiple testing using the Benjamini–Hochberg (BH) procedure (Benjamini and Hochberg, 1995).

Augustus v3.3.3 (Stanke et al., 2006) was utilized to predict protein coding genes from the assembled transcripts using the *Acyrthosiphon pisum* (pea pahid) training set. Through use of a Trinity provided script, the GATK v.3.8 pipeline for variant calling (Van der Auwera et al., 2013) was applied between the transcripts from the biotypes. Variants were accepted as true if they possessed an FS score above 30 (Phred-scaled *p*-value using Fisher's exact test to detect strand bias) and a QD score <2 (Variant Confidence/Quality by Depth). Variants were also required to be present in all 3 biological replicates of one biotype and absent in all 3 biological replicates of the other biotype.

### Analysis of *DNMT* and *TET* Expression

For *DNMT* gene expression analyses, 20 apterous aphids were collected in triplicate for each biotype (3 × *n* = 60) and their heads were removed with a liquid nitrogen-cooled scalpel by cutting carefully posterior to the prothorax (Figure S1) and RNA was extracted as previously described (Burger et al., 2017). cDNA synthesis was performed using the iScript™ cDNA Synthesis kit (BioRad) in accordance with the provided protocol, applying 350 to 400 ng of total RNA as template per 20 μl reaction.

For the host-shift experiment, RNA was isolated from apterous aphid whole-body homogenates prepared using a micro-pestle in liquid nitrogen cooled Eppendorf tubes. Each treatment was represented by three biological replicates consisting of 30 aphids each (*n* = 90). The frozen aphids were ground with micro-pestles and RNA was extracted using RNeasy Mini Kit (Qiagen), following the manufacturers recommended protocol for insect material. cDNA synthesis was performed using SensiFAST cDNA Synthesis Kit (Bioline), with 200 ng of input RNA.

Primer pairs for RT-qPCR (Table S2) were designed using Primer3 from the CDS regions of the RWA sequenced *DNMTs* and *TET* to yield products of between 100 bp and 200 bp in size. Primers were used in a primerBLAST analysis against the assembled RWA SAM biotype reference genome (GCA_001465515.1) to ensure they only matched the *DNMT* and *TET* genes from which they were designed. The relative expression of *DNMT1, DNMT2*, and *DNMT3* (in sampled aphid heads of the RWA biotypes SA1 and SAM), as well as the relative expression of *DnTET* (whole aphids of RWA biotypes SA1 and SAM that underwent host-shifts) was quantified as previously described (Burger et al., 2017). All samples and standards were quantified in triplicate along with a no template control as a measure of contamination. A five point, two times serial dilution of a zero-hour SA1 sample was used to generate quantification standards. The relative expression of *DnDNMT3* and *TET* were calculated using Pfaffl's mathematical model (Pfaffl, 2001) for each time point (0, 6, and 48 h). A CFX96 Real-Time System (Bio-Rad) was used to perform the real-time PCR analysis. Each reaction started with a denaturation step at 95°C for 3 min, followed 40 cycles of amplification, consisting of a denaturation step at 95°C for 10 s, an annealing step at the relevant temperature for each primer set (Table S2) for 30 s, and an extension step at 72°C for 30 s. A melt curve analysis was also performed for each reaction, to verify the absence of non-specific amplification: The incubation temperature was increased in 5 s intervals, 0.5°C at a time, from 65 to 95°C. The ribosomal genes *L27* and *L32* were used as reference genes as they have previously been shown to be constitutively expressed, respectively, in RWA and the pea aphid (Shakesby et al., 2009; Sinha and Smith, 2014).

### Measuring DNMT Protein Activity

For the extraction of aphid protein, three replicates of 150 apterous aphids (*n* = 450) of biotypes SA1 and SAM were collected, flash-frozen and stored at −80°C until use. A micropestle was used to grind aphids into a fine powder, to which 100 μl phosphate buffered saline (50 mM NaH_2_PO_4_, 50 mM Na_2_HPO_4_ and 150 mM NaCl, pH 7.5), 10 μl phenylmethylsulphonyl fluoride (1 mM) and 10 μl dithiothreitol (1 mM) were added. Homogenized mixtures were centrifuged at 15 000 rpm (4°C) for 10 min to pellet the cell debris and the resulting supernatant was transferred to a clean Eppendorf tube. Protein concentrations were quantified using the Bradford protein assay (Bradford, 1976) with Bovine Serum Albumin as standard (BioRad, USA), and the Glomax®-Multi Detection plate reader (Promega, USA) as described by Rylatt and Parish (1982).

DNA methyltransferase protein activity was quantified following the guidelines provided with Abcam's colourimetric DNMT Activity Quantification kit (Abcam, UK), and using the maximum recommended amount of nuclear extract, 5 μl (ranging from 7.69 to 10.96 μg, standardized using the formula below) of each of the three biological replicates per biotype (*n* = 3). DNA methyltransferase activity in OD/h/μg (optical density/hour/microgram) was calculated using the formula below.

$$\begin{array}{l} \text{Protein activity }=\frac{\left(\text{Sample OD }-\text{ Blank OD}\right)}{\left[\text{Protein amount }(\text{ug})\text{ x hour}\right]}\text{ x 1000} \end{array}$$

An ANOVA was performed to test for significant differences between the sample means, with the level of significance set at *p* ≤ 0.05.

### Quantifying Levels of Global Methylation (5mC) and Hydroxymethylation (5hmC)

Global levels of methylation were determined utilizing a colourimetric Methylated DNA Quantification kit (Abcam) using 150 ng DNA of the three biological repeats per biotype (*n* = 3). A slight modification of the protocol was followed in the “methylation capture” section, whereby incubation of DNA and diluted capture antibody was performed for 15 h at room temperature in the dark to allow for optimal antibody binding, as opposed to 1 h at room temperature. The final plate incubation, after addition of the developer solution, was carried out for the maximum recommended time of 10 min. Absorbance at 450 nm was read in triplicate (*n* = 9) within five min of adding the stop solution, using the Glomax®-Multi Detection System. Relative methylation levels were calculated for each sample using the following formula:

$$\begin{array}{l} Relative\;5mC\;\%\;\\ =\;\frac{\left(Sample\;OD\;-\;Negative\;control\;OD\right)/S}{\left(Positive\;control\;OD\;-\;Negative\;control\;OD\right)\;x\;2/P}\;x\;100 \end{array}$$

where 5mC is 5-methylcytosine, OD is optical density, S is the amount of sample DNA in ng and P is the amount of positive control in ng. An ANOVA was performed to test for significant differences between the sample means, with the level of significance set at *p* ≤ 0.05.

Global hydroxymethylation levels were quantified using a colourimetric Hydroxymethylated DNA Quantification kit (Abcam), in accordance with the provided protocol. Freshly extracted DNA sample from each biotype were loaded in triplicate (*n* = 3), and standardized using the formula below (refer to S, the amount of sample DNA). The final plate incubation was carried out for 10 min, where after absorbance at 450 nm was read using the Glomax®-Multi Detection System. Relative hydroxymethylation levels were calculated for each sample using the following formula:

$$\begin{array}{l} Relative\;5hmC\;\%\;\\ =\;\frac{\left(Sample\;OD\;-\;Negative\;control\;II\;OD\right)/S}{\left(Positive\;control\;OD\;-\;Negative\;control\;II\;OD\right)\;x\;5/P}\;x\;100 \end{array}$$

where 5hmC is 5-hydroxymethylcytosine, OD is optical density, S is the amount of sample DNA in ng and P is the amount of positive control in ng. An ANOVA was performed to test for significant differences between the sample means, with the level of significance set at *p* ≤ 0.05.

### Statistical Analysis

Microsoft Excel (2010)/XLSTAT Premium (Addinsoft Inc. USA) were used for the statistical analysis, and SigmaPlot (2001) was used to plot graphs showing the average readings and standard deviation. An ANOVA was performed to test for significant differences between the sample means, with the level of significance set at *p* ≤ 0.05. The model assumptions of ANOVA (i.e., homoscedasticity and normality of the residuals), were tested for using Levene's test and the Shapiro-Wilk test, respectively (significance set at *p* ≤ 0.05 for both tests). If the ANOVA null hypothesis—that the means of the treatment groups are equal—was rejected, a Fisher's LSD test was then performed.

### Whole Genome Bisulfite Sequencing (WGBS) and Analysis

A total of 100 apterous female aphids of South African RWA biotypes SA1 and SAM were used for DNA extraction performed as described previously (Burger and Botha, 2017) (GenBank ID GCA_001465515.1; BioProject PRJNA297165). Three independent biological repeats of each biotype were conducted of each biotype (*n* = 3). Samples consisting of 2 μg DNA, of both RWA biotypes SA1 and SAM were submitted to Macrogen Inc., South Korea for bisulfite treatment, library preparation and sequencing.

DNA samples were treated with the EZ DNA Methylation Lightning kit (Zymo Research) and used to construct the sequencing library utilizing the TruSeq DNA Methylation Library Kit™ (Illumina) (*n* = 1) or Accel-NGS® Methyl-Seq (Swift Biosciences) for Illumina (*n* = 2), 5′ tags were generated through random priming, followed by selective 3′ tagging. Illumina P7 and P5 adapters were ligated through amplification to the 5′ and 3′ ends, respectively. The Illumina HiSeq X platform was used to sequence the bisulfite treated samples.

The obtained sequencing data was analyzed for quality using FastQC (Andrews, 2010). After inspecting the adapter content, per base sequence content, and per base sequence quality, Trimmomatic (Bolger et al., 2014) was used to remove adapter sequences and trim the paired-end reads for quality. A sliding window over 15 bp was used to trim for a quality score of 20, along with a headcrop of 10. The Illuminaclip parameter was used to search for and remove adapter sequences from the reads. After trimming, all reads were filtered for a minimum read length of 40 bp.

The Bismark software program (Krueger and Andrews, 2011) was used to analyze the methylation status of the trimmed and filtered sequence reads. Using the RWA SAM biotype reference genome (GenBank ID GCA_001465515.1; BioProject PRJNA297165), the observed over expected number of cytosine bases for each methylation context was calculated as follows:

$$\begin{array}{l} C_{p}G_{\frac{O}{E}}=\frac{F_{C_{p}G}}{F_{C}.F_{G}}\\ CHG_{\frac{O}{E}}=\frac{F_{CAG}+F_{CTG}+F_{CCG}}{3\left(F_{C}.F_{1-G}{.F}_{G}\right)}\\ CHH_{\frac{O}{E}}=\\ \frac{F_{CAA}+F_{CAT}+F_{CAC}+F_{CTA}+F_{CTT}+F_{CTG}+F_{CGA}+F_{CGT}+F_{CGG}}{9\left(F_{C}{.2F}_{1-G}\right)} \end{array}$$

Where *F* represents the frequency of the subscripted nucleotide/dinucleotide/trinucleotide, in the reference genome. As a reference genome is not available for biotype SA1, the calculations were only performed for biotype SAM.

The R-package DSS-single (Wu et al., 2015) was used to calculate which genes are significantly differentially methylated (*p*-value < 0.05) between SA1 and SAM from the WGBS data. For the analysis, only genic CpG loci, with at least a ten times coverage in both biotypes, across all three repeats were considered. This amounted to 613,730 CpG sites. A Wald test (Wald, 1943) was conducted for differentially methylated loci with the DMLTest function. The optional “smoothing” algorithm of this function, which uses methylation data of nearby loci to generate “pseudo replicates” is only recommended for datasets where methylation loci are dense (Feng et al., 2014). Due to the high AT content and low methylation levels in RWA, methylation loci are sparse and the “smoothing” algorithm was not employed. The CallDMR function was then used to identify differentially methylated regions using information from the differentially methylated loci, such as the number of CpG sites in a region and the percentage of sites in a region scored as significant. This information is used to calculate a combined score for each region, referred to as an area statistic, which can be used to sort regions based on the degree of differentiation in CpG methylation (Wu et al., 2015). The Blast2GO suite (Conesa et al., 2005, Conesa and Götz, 2008) was used to search for the gene ontologies (GO) and KOG terms (Burger and Botha, 2017) of genes containing a differentially methylated region.

## Results

### Classes of DNA Methyltransferases in RWA

**Figure 1:**
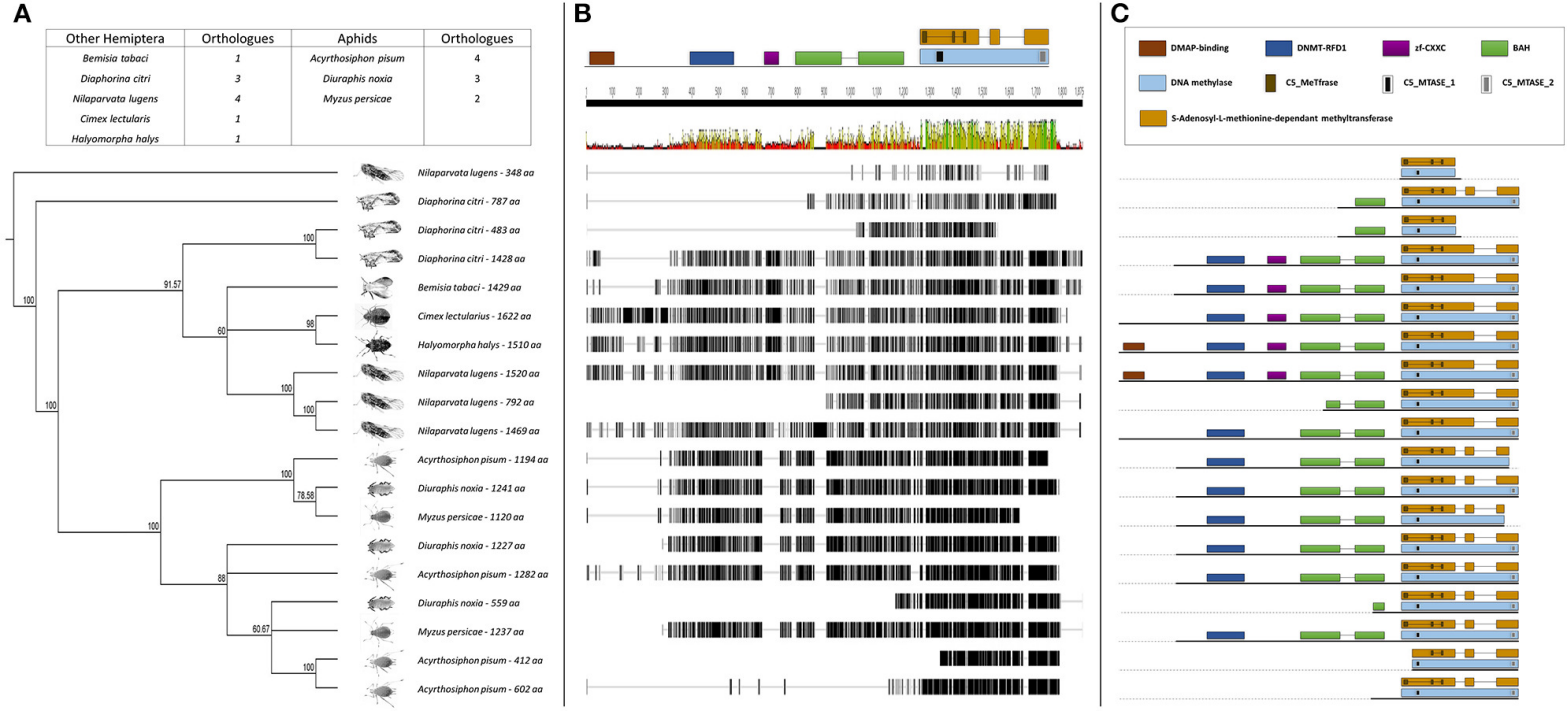
**(A)** Phylogenetic analysis of the DNMT1 amino acid sequences from eight Hemipterans species using MAFFT v7.4 and Paup v4.0a136. Included in the analysis are *Acyrthosiphon pisum, Bemisia tabaci, Cimex lectularius, Diaphorina citri, Diuraphis noxia, Halyomorpha halys, Myzus persicae*, and *Nilaparvata lugens*; **(B)** Comparison of the *DNMT1* gene sequence from the different hemipteran species in **(A)**, showing the level of conservation on gene sequence level; and **(C)** Comparison of the conservancy on functional motifs within the *DNMT1* gene sequence from the different hemipteran species in **(A)**. Indicated are functional and/or structural motifs.

**Figure 2:**
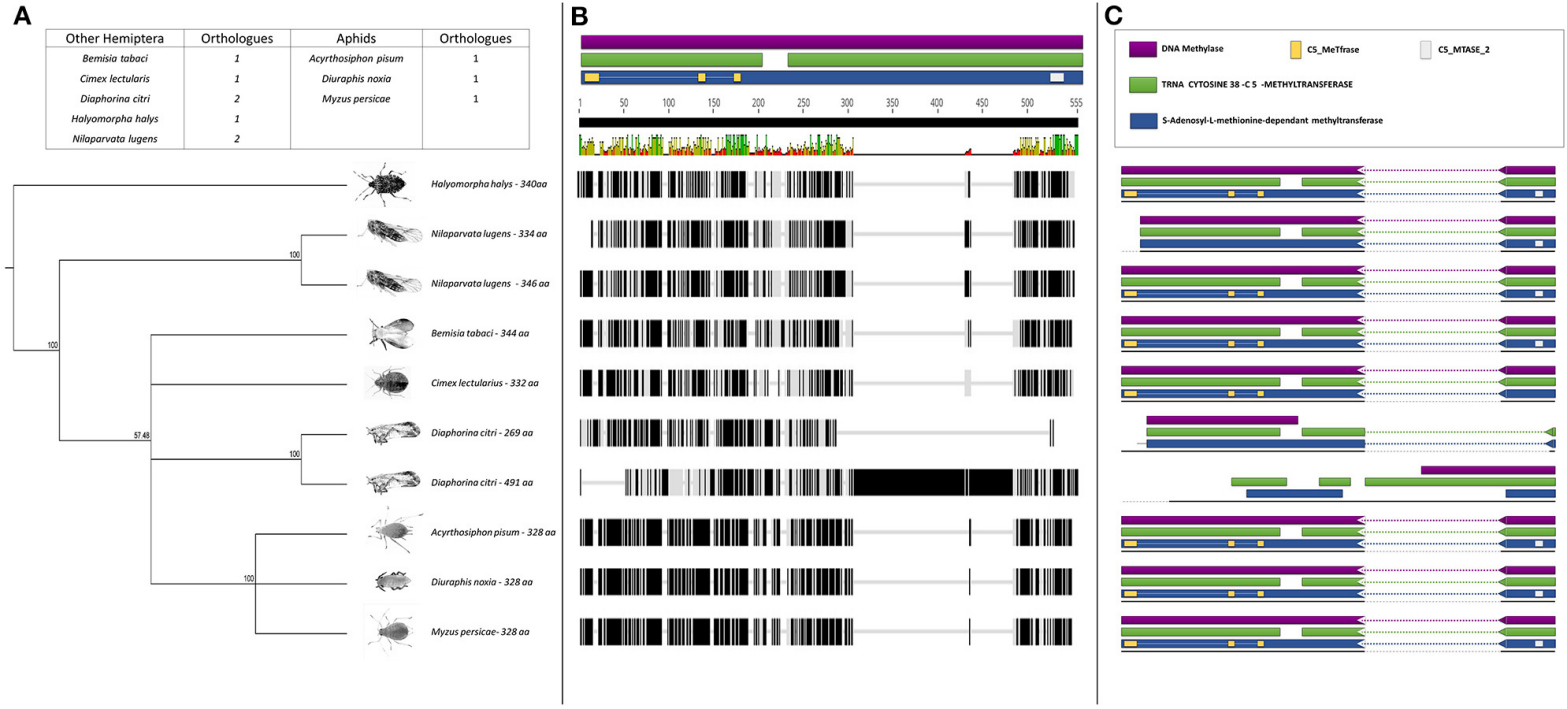
**(A)** Phylogenetic analysis of the DNMT2 amino acid sequences from eight Hemipterans species using MAFFT v7.4 and Paup v4.0a136. Included in the analysis are *Acyrthosiphon pisum, Bemisia tabaci, Cimex lectularius, Diaphorina citri, Diuraphis noxia, Halyomorpha halys, Myzus persicae*, and *Nilaparvata lugens*; **(B)** Comparison of the *DNMT2* gene sequence from the different hemipteran species in **(A)**, showing the level of conservation on gene sequence level; and **(C)** Comparison of the conservancy on functional motifs within the *DNMT2* gene sequence from the different hemipteran species in **(A)**. Indicated are functional and/or structural motifs.

**Figure 3:**
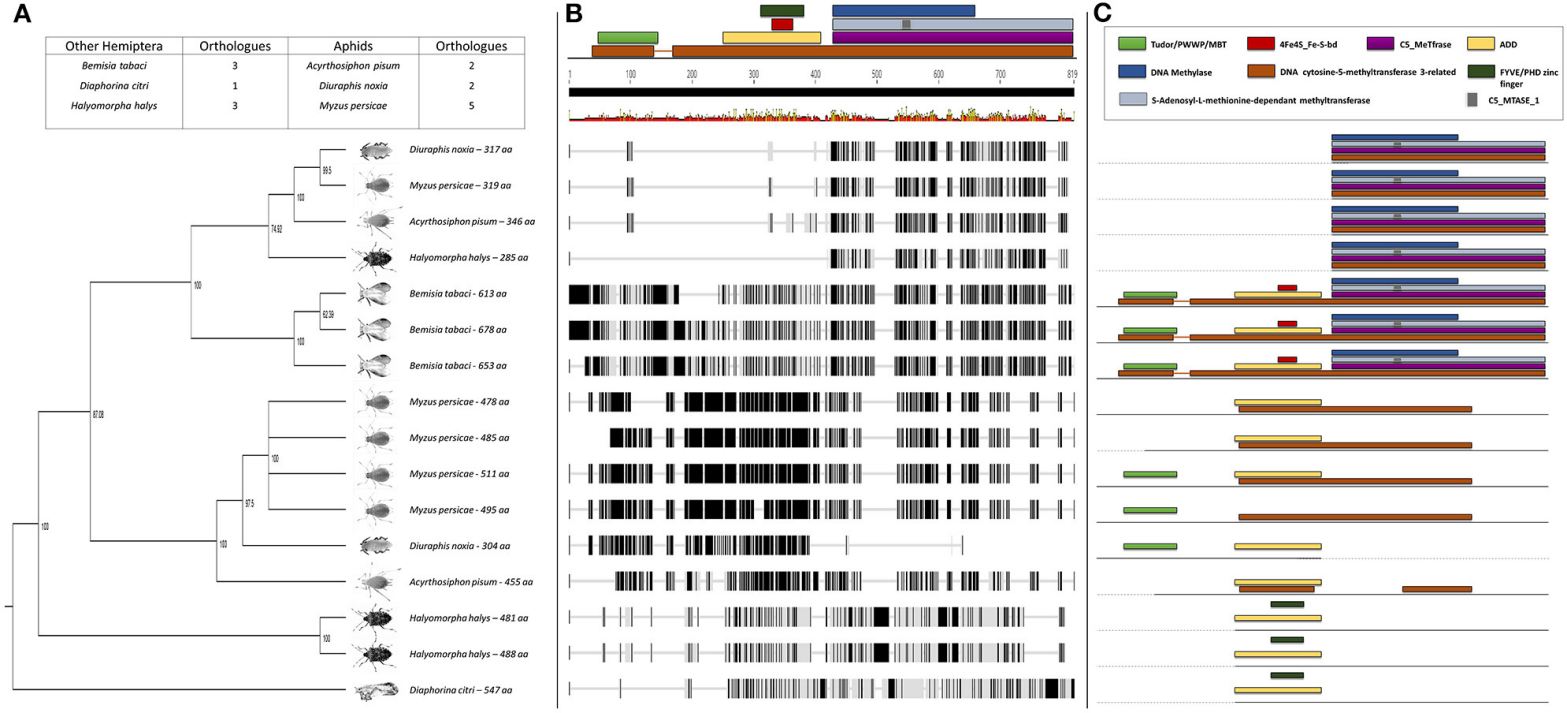
**(A)** Phylogenetic analysis of the DNMT3 amino acid sequences from eight Hemipterans species using MAFFT v7.4 and Paup v4.0a136. Included in the analysis are *Acyrthosiphon pisum, Bemisia tabaci, Cimex lectularius, Diaphorina citri, Diuraphis noxia, Halyomorpha halys, Myzus persicae*, and *Nilaparvata lugens*; **(B)** Comparison of the *DNMT3* gene sequence from the different hemipteran species in **(A)**, showing the level of conservation on gene sequence level; and **(C)** Comparison of the conservancy on functional motifs within the *DNMT3* gene sequence from the different hemipteran species in **(A)**. Indicated are functional and/or structural motifs.

The BLASTp analysis performed using the insect DNMTs against the RWA proteins, revealed three DNMT subfamilies. Comparison of the DNMTs of RWA with other aphids (i.e., *A. pisum* and *Myzus persicae*), as well as with other distant hemipteran species (i.e., *Bemisia tabaci, Cimex lectularius, Diaphorina citri, Halyomorpha halys*, and *Nilaparvata lugen*) confirmed that as with other hemipterans, RWA have three DNMT subfamilies of genes, i.e., *DNMT1, DNMT2, DNMT3* (Figures 1–3). With the DNMT1 and DNMT3 sequences, those from RWA were mostly similar to that of *M. persicae* and then *A. pisum*, than to the other hemipterans included in the study. Whereas, those from *N. lugens* (the brown planthopper) and *D. citri* (Asian citrus psyllid) the most distant from the plant aphids. In the case of DNMT2, the separation was less distinct. These observations were strongly supported by bootstrap values.

Further sequence analysis revealed that the RWA had two homologs of each of the DNMT1s (DNMT1a and DNMT1b) and DNMT3s (DNMT3a and DNMT3b) (Figures 1A, 3A), but only a single DNMT2 (Figure 2B) protein. The RWA DNMTs contained several functional motifs that were recognizable and contributing to the ascribed function, all shared between the plant aphids (Figures 1C, 2C, 3C).

To assess whether the DNMTs differ between RWA biotypes, an alignment of the DNMT sequences obtained between the SA1 and SAM biotypes was conducted which revealed 36 SNPs between the biotypes (Figures S2–S7).

We used the transcriptome data to investigate the expression pattern of known methylation genes in RWA biotypes SA1 and SAM (Figures 5, 6). We again found the full complement of DNA methyltransferases which was expressed in different transcript levels, with minimal differences in *DnDNMT1* and *DnDNMT2* between the RWA biotypes, with fewer *DnDNMT3* transcripts in the more virulent SAM (9.51 ± 6.9) than in the less virulent SA1 (31.45 ± 2.4) (*p* = 0.006).

To measure whether the observed SNPs had any bearing on gene expression between less and more virulent RWA biotypes, the expression of aphid head *DNMTs* among the RWA biotypes was also investigated. Biotype SAM's *DNMT1* expression was higher than that measured in biotype SA1, but the difference in expression was not significant (*p*-value = 0.416 for *L27* and 0.362 for *L32*), as was the expression of *DNMT2* (Figure 5A). The expression of *DNMT3* however showed the most inter-biotype variation of the three *DNMT* subfamilies, and revealed that the *DNMT3* expression levels of SAM (most virulent aphid biotype) were significantly lower than that measured in SA1 (Fisher's LSD test; *p*-value of ≤ 0.1).

To establish whether the difference in the DNMT gene expression equates into measurable differences in total DNMT protein activity, the DNMT protein activity was determined (Figure 5B). The concentration of DNMT protein activity within the biotypes ranged from 44.80 to 53.54 OD/h/μg, with biotype SAM exhibiting the lower DNMT protein activity of the biotypes. However, the DNMT protein activity levels did not differ significantly between the biotypes (*p* ≤ 0.05).

### Sequence Analysis and Expression of *DnTET* in RWA Biotypes

**Figure 4:**
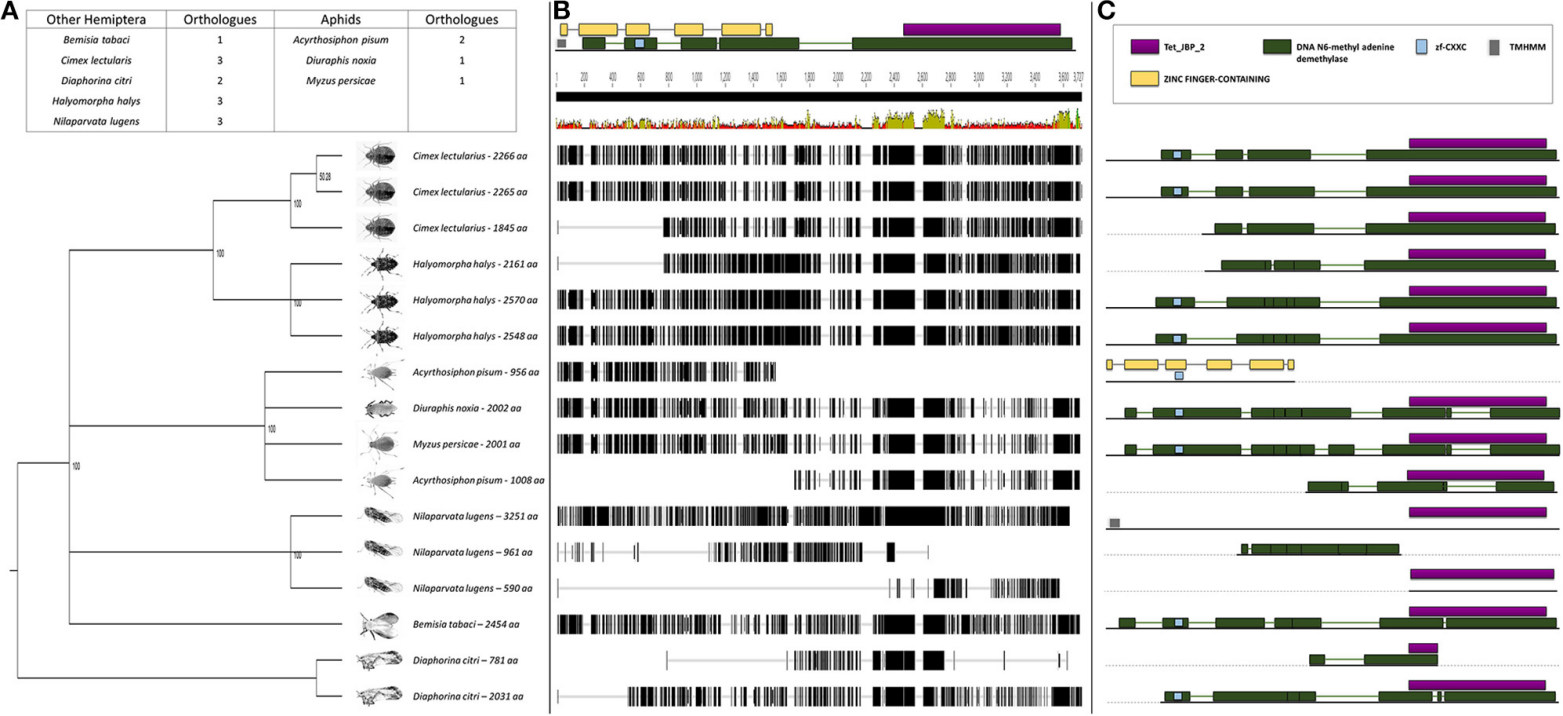
**(A)** Phylogenetic analysis of the N6-methyl adenine demethylase (TET-like) amino acid sequences from eight Hemipterans species using MAFFT v7.4 and Paup v4.0a136. Included in the analysis are *Acyrthosiphon pisum, Bemisia tabaci, Cimex lectularius, Diaphorina citri, Diuraphis noxia, Halyomorpha halys, Myzus persicae*, and *Nilaparvata lugens*; **(B)** Comparison of the *N6-methyl adenine demethylase* (*TET-like*) gene sequence from the different hemipteran species in **(A)** showing the level of conservation on gene sequence level; and **(C)** Comparison of the conservancy on functional motifs within the *N6-methyl adenine demethylase* (*TET-like*) gene sequence from the different hemipteran species in **(A)**. Indicated are Tet_JBP_2, DNA N6-methyl adenine demethylase, zf-CXXC, TMHMM, and Zinc finger-containing motifs.

To shed light on the observed difference in global demethylation but not methylation levels, the *TET (N6-methyl adenine demethylase)* genes responsible for oxidation within the methylation pathway were studied (Wojciechowski et al., 2014). We were able to isolate and sequence the *DnTET* ortholog from RWA. Comparison of the DnTET (N6-methyl adenine demethylase) protein sequences in RWA with other aphids (i.e., *A. pisum, M. persicae)* and with other distant hemipteran species (i.e., *B. tabaci, C. lectularius, D. citri, H. halys*, and *N. lugen*) confirmed that all these species have recognizable TET-like sequences in their genomes (Figure 4). Clustering of the sequences group the aphids closer to each other than to the other hemipterans, with strong bootstrap support (Figure 4A). Further analysis revealed that unlike *A. pisum* and some of the other hemipteran species, RWA has only a single form of TET (Figure 4B), which contain several functional motifs including DNA N6-methyl adenine demethylase (Figure 4C).

We also used the transcriptome data to investigate the expression pattern of the *TET (N6-methyl adenine demethylase)* genes responsible for oxidation within the methylation pathway in RWA biotypes SA1 and SAM (Figure 6C). We found more *DnTET* transcripts in the more virulent SAM (5.81 ± 0.38) than in the less virulent SA1 (2.59 ± 2.3) (*p* = 0.014).

**Figure 5:**
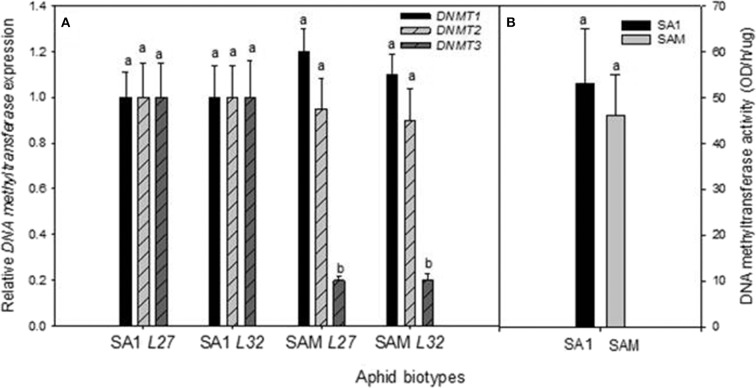
**(A)** Comparison of the average relative expression (R mean) of *DNMTs* in South African RWA biotype heads. Fold changes in expression are shown relative to the SA1 samples, the expression of which was set at 1. *DNMTs* expression is presented when normalized against the reference genes *L27* and *L32*, respectively, and the error bars indicate standard deviation. Different alphabetical letters indicate statistical significance (*p* ≤ 0.05). **(B)** DNA Methyltransferase protein activity (OD/h/μg) measured in South African RWA biotypes SA1 and SAM, with error bars indicating the standard deviation.

**Figure 6:**
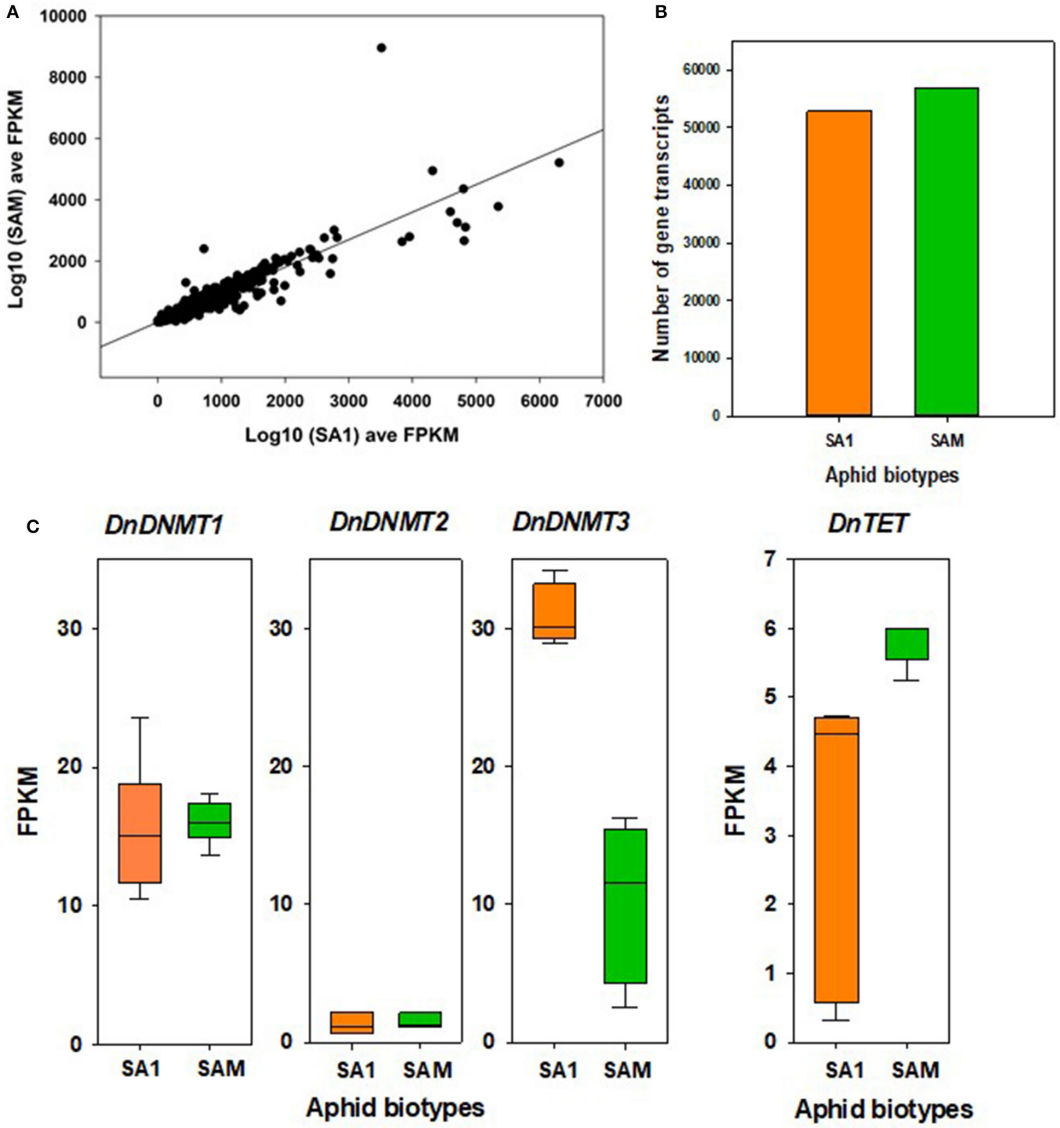
Differential gene expression between RWA biotypes SA1 and SAM. **(A)** SA1 and SAM gene expression as log10 fragments per kilobase of transcript per million mapped reads (FPKM) average over three biological replicates for transcripts retained for differential expression (DE) analysis with edgeR (*n* = 64,214). Benjamini-Hochberg (BH) corrected *p* > 0.05 and absolute fold change [FC] > 1.5). **(B)** Comparison of number of transcripts expressed in SA1 and SAM. **(C)** Significant differences were observed in the expression of *DnDNMT3* and *DnTET* in SA1 and SAM; edgeR; BH corrected *p* > 0.05 and absolute fold change [FC] > 1.5.

### Expression of DNA Methylation Genes During Feeding Studies

**Figure 7:**
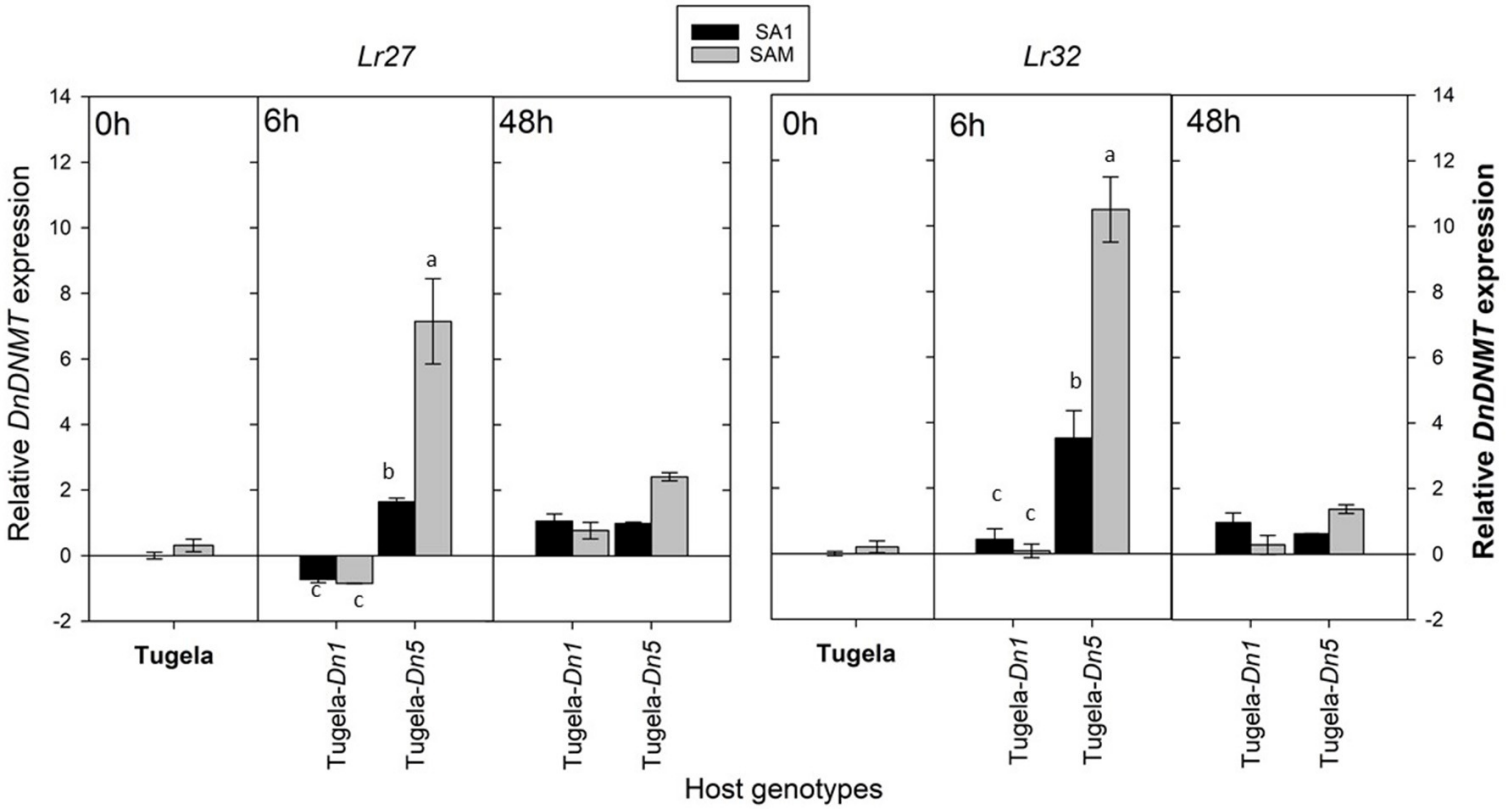
A comparison of the average relative expression (R mean) of *DnDNMT* of South African RWA biotypes after transfer to different host plants after 6 and 48 h of feeding. Fold changes in expression are shown relative to the SA1 samples, the expression of which was set at 1. *DnDNMT* expression is presented when normalized against the reference genes *L27* and *L32* respectively, and the error bars indicate standard deviation. Different alphabetic letters indicate significant differences (*p* ≤ 0.05).

To assess further whether the sequenced *DnDNMT* and *DnTET* genes expressed in RWA biotypes SA1 and SAM, differ when challenged with different host plants, the RWA biotypes were reared on susceptible host plants (*n* = 3) (Table 1) and then moved to two resistant wheat cultivars (Tugela-*Dn1* and Tugela-*Dn5*). The lines were selected based on the fact that they are near isogenic and differ only with regard to the resistance gene present (i.e., *Dn1* expressing antibiosis—harmful to the aphid and *Dn5* expressing antibiosis and antixenosis—non-palatable) (Figure 7). The measured *DnDNMT* expression increased significantly in both aphid biotypes when challenged with a new feeding environment (i.e., *Dn5* expressing both antibiotic and antixenotic) (*p* ≤ 0.05). The relative expression almost doubled in the less virulent biotype SA1, but even more in the more virulent biotype SAM (± six-fold *Lr27*; ±11-fold *Lr32*) within 6 h after host-shifting.

**Table 1:** Relative expression of *DNMT3* and *TET* in RWA biotypes SA1 and SAM when feeding on a susceptible cultivar SST.

**Relative gene expression**	**Aphid biotype**			
	**SA1**		**SAM**	
**Reference genes**	***Lr27***	***Lr32***	***Lr27***	***Lr32***
DNMT	0.852 ± 0.126	0.734 ± 0.084	1.308 ± 0.531	0.749 ± 0.221
TET	0.758 ± 0.114	0.652 ± 0.072	1.222 ± 0.304	0.721 ± 0.223

*Lr27 and Lr32 was used as reference genes*.

**Figure 8:**
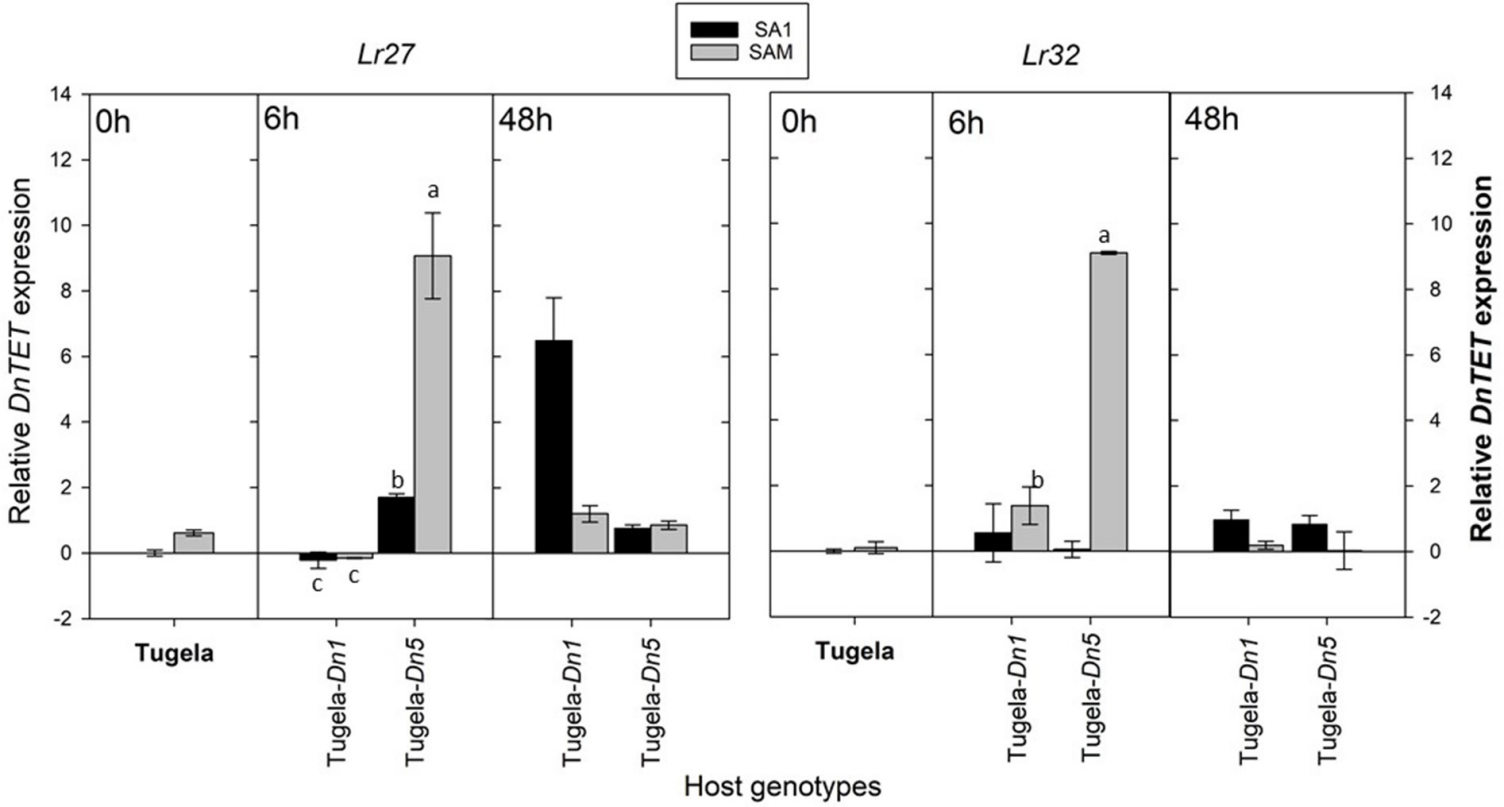
A comparison of the average relative expression (R mean) of *DnTET* of South African RWA biotypes after transfer to different host plants after 6 and 48 h of feeding. Fold changes in expression are shown relative to the SA1 samples, the expression of which was set at 1. *DnTET* expression is presented when normalized against the reference genes *L27* and *L32*, respectively, and the error bars indicate standard deviation. Different alphabetic letters indicate significant differences (*p* ≤ 0.05).

To assess whether the sequenced *DnTET* gene was also differentially expressed in RWA, biotypes SA1 and SAM during feeding after host-shifting, the expression of *DnTET* was also measured (Figure 8). The measured *DnTET* expression increased slightly but not significantly after 6 h in the less virulent SA1 biotypes when challenged with the *Dn5* wheat line (antibiotic and antixenotic) (*p* > 0.05), but significantly in SA1 after 48 h when challenged with the antibiotic wheat line Tugela-*Dn1* (*Lr27*) (*p* ≤ 0.05). In contrast, the *DnTET* expression of virulent biotype SAM remained the same when challenged with the antibiotic wheat line Tugela-*Dn1*, but increased significantly within the first 6 h after host-shifting from the susceptible Tugela to the wheat line containing the *Dn5* resistance gene (*p* ≤ 0.05).

### Global Methylation and Hydroxymethylation Quantification

**Figure 9:**
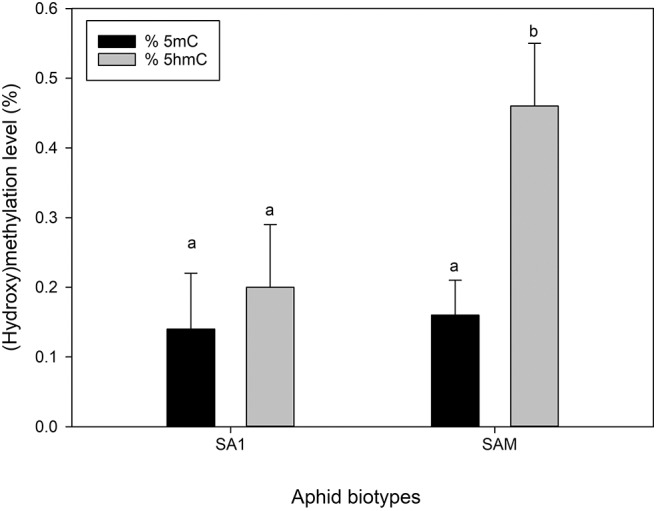
Comparison of global 5 mC (black) and 5 hmC (gray) levels of the South African RWA biotypes. The error bars indicate standard deviation and different alphabetic letters indicate significant differences (*p* ≤ 0.05).

To quantify the global levels of methylation (5mC) and dehydroxymenthylation (5hmC), antibodies specific to these (i.e., 5mC and 5hmC) were used. The use of the 5mC antibody revealed similar levels of global methylation between the less virulent SA1 and more virulent SAM biotype, with the measured levels, ranging between 0.14 and 0.16% (Figure 9). The hydroxymethylation levels, however differed significantly ranging from 0.12 to 0.46% (Figure 9), with biotype SA1 displaying the lowest, and biotype SAM displaying the highest 5hmC levels, respectively (*p* ≤ 0.05).

### Whole Genome Bisulfite Sequencing

The whole genome bisulfite sequencing produced a total of 6,846,597,083 raw reads for SA1 and 7,397,965,699 raw reads for SAM, respectively, of which a total of 70,861,462 bases (SA1) and 74,073,939 bases (SAM) were methylated, which represents 1.126 ± 0.321% (SA1) and 1.105 ± 0.295% (SAM) methylation in the genome (Tables S3, S4). The sequence reads were analyzed for contexts of DNA methylation within the genome (Table S5) and the results revealed that RWA has methylation in all contexts (CpG, CHG, and CHH), with the majority of methylation within the CpG context (±5.19%), while the other contexts show much lower levels of methylation (CHG—±0.27%; CHH—±0.34%). The reads were then subjected to quality analysis, aligned and mapped to the RWA biotype SAM reference genome (GenBank ID GCA_001465515.1; BioProject PRJNA297165). Of the methylated reads, most of the methylation was located in genic regions (±1.58%), but intergenic methylation was also present (±0.808%) (Table S6). Methylation was evenly distributed between both strands, with the top strand only containing 0.02% more methylated calls than the bottom strand (Table S7). Within genes exonic regions were found to be overall more methylated (±0.56%) than the intronic regions (Table S8), and the most represented context of methylation (i.e., CpG, CHG, or CHH) was in the CpG context, followed by the CHG context, with the least in the CHH context (Table S9).

**Figure 10:**
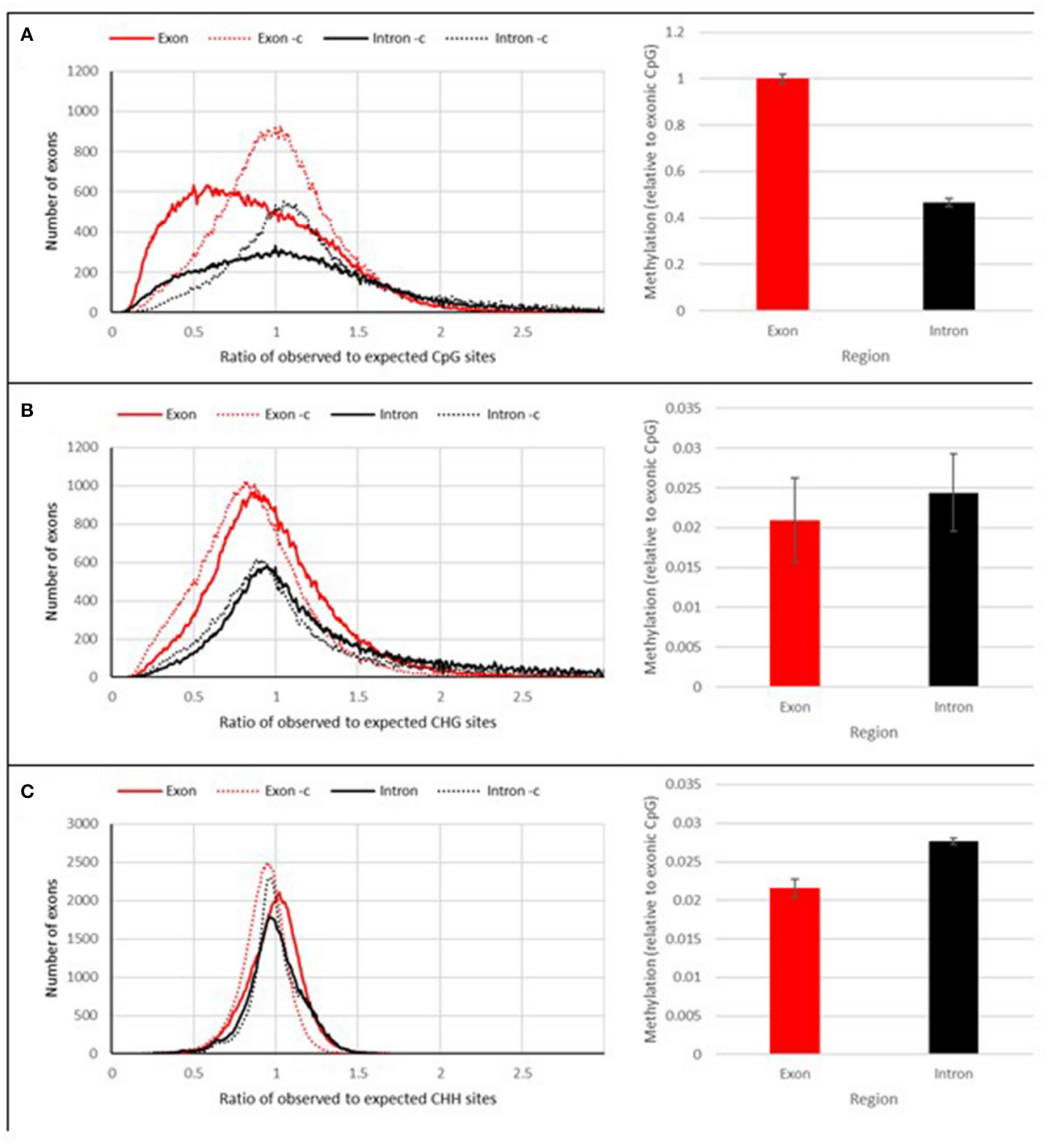
DNA methylation context. The calculated observed over expected number of cytosine bases in the genome of RWA. **(A)**, CpG(_O/E_) **(B)**, CHG(_O/E_), **(C)** CHH(_O/E_).

Using the SAM biotype reference genome, the observed over expected number of cytosine bases were also calculated (Figure 10). This is commonly seen for the CpG context, denoted as *CpG*_*O*/*E*_ (Hunt et al., 2010), with the data on all three contexts of cytosine methylation available from the Bismark pipeline, the other, often overlooked *CHG*_*O*/*E*_ and *CHH*_*O*/*E*_ ratios were also included in this study. After calculating these ratios, it was revealed that the observed CpG context, unlike with the other contexts was lower than expected (Figure 10). This is particularly so for the observed CpG context in exonic regions (Figure 10A).

**Figure 11:**
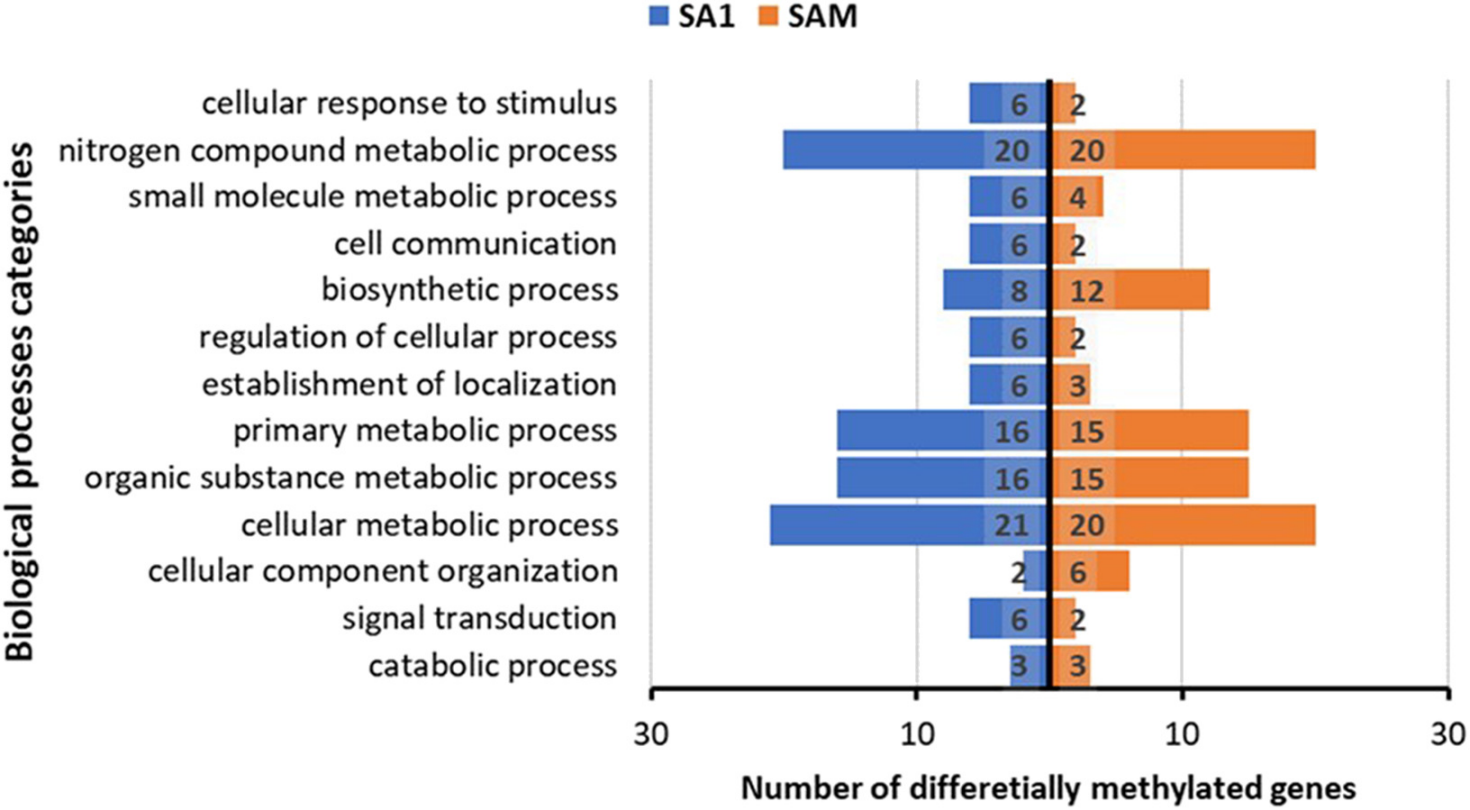
Differentially methylated genes (DMEs) differ in numbers between the less virulent biotype SA1 and more virulent SAM when group in broad biological process categories.

After analysis, we identified 40 differentially methylated genes (DMEs) when we compared the genes that were differentially methylated between the less and more virulent biotypes (Figure 11; Figures S8A1-3, B1-9, C1-13). Even though based on broad functional categories, the differences between the least virulent SA1 and most virulent SAM seems minimal (Figure 11), further analyses of their involvement into biochemical pathways revealed that these DMEs had distinctly different predicted functions (even when involved within the same pathway, Figures S8A1-3). Interesting examples include the selective methylation of genes in more virulent SAMs, but not SA1, which include include DMEs involved in the irinotecan metabolism (Figure S8A3); metabolism of cytotoxicity by cytochrome P450 (Figures S8C10, C11); steroid hormone biosynthesis (Figure S8C2) and wax biosynthesis (Figure S8C).

## Discussion

Integrated pest management programs against RWA depend heavily on the breeding of wheat cultivars that provide resistance (Tolmay et al., 1997; Smith and Clement, 2012; Botha, 2013; Sinha and Smith, 2014). The effectiveness of these cultivars, however, is often short-lived as aphids overcome the resistance they impart (Botha et al., 2005, 2010; Tagu et al., 2008; Sinha and Smith, 2014). Understanding how new aphid biotypes develop, as well as the mechanisms they employ to exert their virulence enabling them to breakdown plant resistance, are of utmost importance if resistant cultivars are to be used to their full potential (Botha et al., 2014a). The availability of the highly virulent mutant RWA biotype (SAM) (Swanevelder et al., 2010), alongside South Africa's naturally occurring biotypes (SA1, SA2, SA3, and SA4) (Walters et al., 1980; Tolmay et al., 2007; Jankielsohn, 2011, 2016) presents a unique opportunity for the study of biotypification. Despite having developed from SA1, which only renders *dn3*-containing cultivars susceptible (Jankielsohn, 2011), SAM has the remarkable ability to overcome the resistance of all the *Dn* genes that have been introduced and/or documented (Botha, 2013; Botha et al., 2014a). SAM thus serves as a model to resolve aphid biotypification.

In the present study, we investigated the DNMT protein family, as they catalyze the covalent addition of a methyl group to the 5′ position of cytosine in the methylation pathway. *DNMT2* seemed the most conserved with only a single form of the protein responsible for stabilizing tRNA and the regulation of protein synthesis in response to environmental cues (Becker and Weigel, 2015). In contrast, *DNMT1* and *DNMT3* with two forms each, are responsible for maintaining and establishing methylation patterns, respectively (Goll and Bestor, 2005; Goll et al., 2006; Jeltsch et al., 2006). Owing to the important role of these proteins in changing methylation patterns, it is not surprising that variations in these genes occur. RWA is also not the only insect showing multiple homologs within a specific DNMT class. Some insect lineages were shown to lack one (e.g., *B. mori* and *T. castaneum*) or two (e.g., *D. melanogaster* and *A.gambiae*) classes of *DNMTs*, while others have multiple homologs (e.g., *A. mellifera, N. vitripennis* and *A. pisum*) within a certain *DNMT* class (Kunert et al., 2003; Marhold et al., 2004; Walsh et al., 2010; Xiang et al., 2010; Glastad et al., 2011; Feliciello et al., 2013).

The limited available literature on aphid *DNMTs* prompted an investigation into the baseline *DNMT* expression (i.e., expression of aphids not challenged with resistance) of South African RWA. It is widely assumed that the insect DNMTs have the same functions as their mammalian orthologs (Wang et al., 2006; Glastad et al., 2014). *DNA methyltransferase 3* (*DNMT3)* was the only gene of which the expression was significantly different between the two RWA biotypes. *DNMT3* has long been known as a *de novo* methyltransferase (Okano et al., 1999; Goll and Bestor, 2005), which establishes new methylation patterns by methylating previously unmethylated sites (Kunert et al., 2003; Schaefer and Lyko, 2007). The *DNMT3* expression of the virulent biotype used in this study, SAM, is down-regulated in comparison to the less virulent biotype, SA1, and this decrease in expression could therefore be advantageous from a virulence perspective.

A role for *DNMT3A* in the facilitation of transcription has also been identified, with *DNMT3A*-dependent methylation of gene bodies promoting transcription by antagonizing polycomb repression (Wu et al., 2010). Although the aphid effector genes are yet to be identified (Botha et al., 2005, 2014b), it is possible that they contain *DNMT3A* binding sites within their gene bodies, and that their transcription could be facilitated by *DNMT3A* binding and subsequent methylation. In the current study, SA1's *DNMT3A* expression, and therefore DNMT3A protein production, is up-regulated in comparison to the more virulent biotype. The fact that SA1 has higher *DNMT3A* expression (and perhaps greater effector protein production) under unchallenged conditions, may provide some insight into why SA1 is the least virulent biotype. Therefore, quantifying the *DNMT3A* expression of aphids challenged by resistance may yield valuable information on *DNMT3A*'s possible involvement in effector transcription.

Other functions of *DNMT3* include its role in the removal of 5mC and 5hmC (Chen et al., 2012, 2013) and a proposed involvement in the maintenance of methylation, by being able to “methylate sites missed by DNMT1 activity” (Jones and Liang, 2009). However, as the *DNMT3*-mediated removal of 5mC and 5hmC is dependent on certain redox conditions (Chen et al., 2012, 2013), and has only been shown to occur *in vitro* (Chen et al., 2012, 2013), it is difficult to draw conclusions regarding the *DNMT3* expression and its potential demethylating and dehydroxymethylating activities in RWA. The DNA methyltransferase 3 protein is assumed to help maintain methylation in densely methylated areas of mammalian genomes (Jones and Liang, 2009).

The hydroxylation of methylated cytosines by TET enzymes, resulting in the formation of 5hmC, is one of various active demethylation mechanisms (Tahiliani et al., 2009; Branco et al., 2012). The initial functional characterization of TETs was performed in mammals, which have three TET enzymes, namely TET1, TET2, and TET3 (Iyer et al., 2009; Tahiliani et al., 2009). In contrast to this, invertebrates possess only a single TET ortholog (Pastor et al., 2013; Wojciechowski et al., 2014), which has been identified in insects containing hydroxymethylation, including *A. mellifera* (Cingolani et al., 2013; Wojciechowski et al., 2014), *T. castaneum* (Feliciello et al., 2013)*, N. vitripennis* (Pegoraro et al., 2016) and *D. melanogaster* (Dunwell et al., 2013). In 2014, Wojciechowski et al. functionally characterized the *A. mellifera* TET ortholog, AmTET, and concluded that, like the mammalian TETs, AmTET is capable of hydroxylating 5mC to form 5hmC. This provided the first evidence that TETs play a similar role in insects, as they do in mammals. The presence of measurable amounts of 5hmC in the RWA biotypes tested, suggests that at least one active demethylation pathway (i.e., hydroxylation of 5mC by TET) is present in RWA, as confirmed in the current study when we sequenced the *DnTET* ortholog. We then studied the expression of this gene in the RWA biotypes after challenging the aphids through differential feeding, as this was previously shown to be perceived as stressful (Burger et al., 2017). Interestingly, when SA1 feeds on wheat with an antibiotic mode of resistance (e.g., Tugela-*Dn1*), an oxidative burst (elevated H_2_O_2_) occurs at the feeding sites (Botha et al., 2014b; Burger et al., 2017), and the expression of *DnTET* more than doubles. Whereas, when SA1 feed on wheat expressing both antibiosis and antixenosis (e.g., Tugela-*Dn5*), not only is the aphid challenged by the elevated H_2_O_2_ but also by volatile substances that make the wheat unpalatable (Botha et al., 2014b), resulting in the tripling of *DnTET* expression. A similar trend is not observed with the expression of *DnTET* in SAM. Biotype SAM feeding, however, is not associated with an oxidative burst or increased peroxidase activity levels, because SAM “avoids” detection by wheat hosts (Botha et al., 2014a).

In the present study, we also studied the epigenome of RWA biotypes SA1 (least virulent SA biotype) and SAM (most virulent SA biotype). The whole-genome bisulfite sequencing indicated that the genomes of these biotypes were globally more methylated (i.e., 1.126 ± 0.321% for SA1; 1.105 ± 0.295% for SAM) than previously reported for insect genomes. For example, the global methylation levels of *A*. *mellifera* (Lyko et al., 2010), *B*. *mori* (Xiang et al., 2010), the ants *Camponotus floridanus* and *Harpegnathos saltator* (Bonasio et al., 2012) and *N. vitripennis* (Beeler et al., 2014) are all between 0.1 and 0.2%.

However, when quantified using the antibody-based methods, the global methylation levels (0.14–0.16%) are in line with other reports of insect methylation. Panikar et al. (2015) investigated adult *D*. *melanogaster* methylation, also through an antibody-based method, found the adult *D*. *melanogaster* genome to be ~0.5% methylated. Russian wheat aphids thus have low, but detectable levels of methylation which are ~0.2 to 0.4-fold of that of the model organism *D. melanogaster*, as measured using the same technique, which allows a more direct comparison. Although other authors have reported lower levels of adult *D. melanogaster* methylation using bisulfite sequencing (0% – Lyko et al., 2000), liquid chromatography tandem mass spectrometry (0.034% – Capuano et al., 2014) and thin layer chromatography (0.05–0.1% – Gowher et al., 2000).

The sequence reads were analyzed for contexts of DNA methylation within the genome and the results revealed that RWA has methylation in all contexts (CpG, CHG and CHH), with the majority of methylation within the CpG context (±5.19%), but still notable methylation in the other contexts (CHG—± 0.27%; CHH—± 0.34%), with most of the methylation located in the genic regions. A similar finding was recently reported by Mathers et al. (2019) in the green peach aphid, *Myzus persicae*. The authors found that exons are highly enriched for methylated CpGs, particularly at the 3′ end of genes. Their findings also alludes to sex-biased differential methylation of genes involved in aphid sexual differentiation.

The model organisms, mice (*Mus musculus*) and zebra fish (*Danio rerio*), showed very low levels of CHH and CHG methylation (1% and lower) when compared to the 74.2% and 80.3% CpG methylation observed, respectively. Low levels of CHG (0.26%) and CHH (0.17%) were also reported for the honeybee (*Apis mellifera*) (Feng et al., 2010). In another insect example, the over expression of DNMT2-like protein in fruit flies (*Drosophila melanogaster*) resulted in observable CpT and CpA methylation (Kunert et al., 2003).

We also wanted to assess whether the DNA strands (top vs. bottom) were methylated equally and found that the top strands were slightly more methylated (0.06%) than the bottom ones. However, interestingly the difference between the methylation of the top and bottom strands were significantly more in the more virulent SAM (0.09%), than SA1 (0.02%). This was not an unexpected finding, as biotype SAM was previously found to exhibit the highest level of hemimethylation (at the external cytosine) when its methylation was investigated using the Methylation-Sensitive Amplification Polymorphism (MSAP) technique (Breeds et al., 2018). Hemimethylated DNA arises during DNA replication, as the newly synthesized daughter strand contains unmodified cytosines (Jeltsch, 2002; Goll and Bestor, 2005). When the levels of 5-hmC were measured using the antibody based method, earlier observations (Breeds et al., 2018) were confirmed, as the levels of 5hmC differed significantly between the aphid biotypes, with SAM much higher than that measured in the less virulent SA1 (*p* < 0.05; Figure 9).

Analysis of the 40 differentially methylated genes (DMEs) revealed that the less and more virulent biotypes had distinctly different DMEs. As previously indicated, DMEs in the more virulent biotype SAM include DMEs involved in the irinotecan metabolism where is it seemingly regulates the conversion between SN-38 and SN38G which regulates secretion. SN-38 produced in the body by carboxylesterase is the active metabolite of irinotecan (Fujita et al., 2015), with its mechanism of action thought to be its interaction with the cleavable complex of DNA and a nuclear protein topoisomerase I. This then results in a blockade of DNA replication (Hsiang and Liu, 1988; Hertzberg et al., 1989) which causes double-strand DNA breakage and cell death. This process may be linked to the metabolism of xenobiotics by cytochrome P450 and ascorbate and aldarate metabolism that may enable SAM to counter its feeding environment better than its parent, the less virulent SA1. In locusts, it was demonstrated that an ascorbate-recycling system in the midgut lumen can act as an effective antioxidant defense in caterpillars that feed on prooxidant-rich foods, emphasizing the importance of a defensive strategy in herbivorous insects based on the maintenance of conditions in the gut lumen that reduce or eliminate the potential prooxidant behavior of ingested phenols (Barbehenn et al., 2001). Interestingly, more virulent SAM also selectively methylates genes associated with steroid hormone biosynthesis and wax biosynthesis, both pathways producing chemicals that affect the physiological processes associated with insect development and insect survival (Niwa and Niwa, 2016). The less virulent SA1 have DMEs associated with the glutathione biosynthesis which may signal stress responses.

Collectively, the results suggest that RWA biotype SAM has a greater capacity to actively methylate/demethylate its DNA than its parent SA1. Thus, it can be concluded with fair confidence that SAM undergoes more methylation/demethylation than its parent biotype SA1. However, the question that remains is whether this ability to actively methylate/demethylate occurs at specific sets of genes depending on the environmental cue/stress RWA is faced with, as opposed to occurring globally (although global, genome-wide demethylation was measured). As gene bodies are the predominant sites of methylation in insects (Zemach et al., 2010; Glastad et al., 2011; Lyko and Maleszka, 2011), it is likely that it is in these regions that methylation will be removed. Also, removal of intragenic methylation of certain genes may alter the transcripts that are produced, by exposing cryptic binding sites or intragenic promoters (Maunakea et al., 2010; Hunt et al., 2013a) and/or affect the splice variants that are produced, through methylation's involvement in alternative splicing (Lyko and Maleszka, 2011; Shukla et al., 2011; Bonasio et al., 2012; Maunakea et al., 2013; Glastad et al., 2014; Yan et al., 2015). As demethylation can occur in a matter of hours (Glastad et al., 2011), the greater capability of SAM to demethylate its genome, may provide SAM with more flexibility to adapt to changing environments, and therefore may underlie SAM's ability to overcome plant resistance. However, this is an aspect that requires further investigation in future.

## Data Availability Statement

The whole epigenome sequencing data from SA1 (accession SRX4643785) and SAM (accession SRX4643786) were deposited to the National Center for Biotechnology Information (NCBI) (GEO SUBMISSION GSE119504) (https://www.ncbi.nlm.nih.gov/gds/?term=diuraphis%20noxia) with Bioproject number PRJNA489432.

## Author Contributions

A-MB conceived and designed the experiments as well as drafted the manuscript. Aphid rearing and sampling was performed by KB and NB. DNMT expression and TET expression was performed by KB and JT respectively, while DNMT and TET sequencing was performed by KB and HS, respectively. Alignments and phylogentic analyses of DNMT and TET sequences were performed by NB while 5mC and 5hmC analyses were performed by KB, NB, and A-MB. Whole genome bisulfite sequencing and its analysis was performed by PP and NB. All authors edited and reviewed the final manuscript.

## Conflict of Interest

The authors declare that the research was conducted in the absence of any commercial or financial relationships that could be construed as a potential conflict of interest.
