# Supplementary material for: DNA Methylation and Demethylation Are Regulated by Functional DNA Methyltransferases and DnTET Enzymes in Diuraphis noxia
Source: Front Genet. 2020 Jun 23;11:452. doi: 10.3389/fgene.2020.00452 (PMC7324797; doi:10.3389/fgene.2020.00452)
Supplement: Supplementary file 1 [file Data_Sheet_1.docx]

**List of Supplemental tables**

**Table S**1. Sequences of primers designed to amplify regions of the *DNMT* (subfamily 1 through 3) and *TET* coding domain sequences, and M13 primers matching regions of the pTZ57R/T vector for sequencing purposes.

| ***Gene target*** | ***D. noxia* gene** | **Forward primer sequence (5’-3’)** | **Reverse primer sequence (5’-3’)** |
| --- | --- | --- | --- |
| ***DNMT* subfamily** |  |  |  |
| 1 | g3935.t2 set 1 | TCATGGTGGGTATACAAAAGT | TTCTGGCCAATATTCATTGTTT |
|  | g3935.t2 set 2 | TCCACTAAACCCTTGACAAG | GTTGGAAAATTCAATGCGTG |
|  | g3935.t2 set 3 | GCTTTTTTGAATGTTCCAGG | ACTTGTGAAGACTGTTTTTGA |
|  | g3935.t2 set 4 | TTCATTGTTTCTTCACTTTCT | AGGTTTTGGCCAATATTTTCA |
| 2 | g9354.t2 | TCAGTGGTATTGGTGGAATG | TTTTGCTGACTGGAAAGTCT |
| 3A | g25709.t2 | TGGTGGTTGAGTGATGATAG | GGGTGTTCAATCTTTGGTTT |
| 3A | g31769.t2 | TGTTGATGATTGCAATGAGAA | TCTGAGTTGTTAATTGTTTGATGT |
| 3B | g175.t2 set 1 | TTGTCTCTCTTTGACGGAAT | GAACAATATACCACTTCCATCTG |
|  | g175.t2 set 2 | TGACTTTTACCGTGTTTGGA | CTTTTTGAAGTGATGGTACCAA |
| ***TET*** | g5037.t2 & g5038.t2 set 1 | ATGAGCGAGGGTGGCG | AACTCGACTCCTCTCCTCCC |
|  | g5037.t2 & g5038.t2 set 2 | GAGCCCTCAATACCCACCAC | AACTCGACTCCTCTCCTCCC |
|  | g5037.t2 & g5038.t2 set 3 | ATGGCTTATAGAAACAAGGGCAAC | AACTCGACTCCTCTCCTCCC |
|  | g5037.t2 & g5038.t2 set 4 | GTACCCACCTCAACAACCTC | CTATTGACATTGGTTCTCCTGGTA |
|  | g5037.t2 & g5038.t2 set 5 | AACAACAGCAGCATCATCAAA | CTATTGACATTGGTTCTCCTGGTA |
|  | g5037.t2 & g5038.t2 set 6 | GATTGCAATTGTTTCCCTACGG | CTATTGACATTGGTTCTCCTGGTA |
|  | g5037.t2 & g5038.t2 set 7 | GCTCACCGAGACTTTCATAACAT | CTATTGACATTGGTTCTCCTGGTA |
|  | g5037.t2 & g5038.t2 set 8 | ATCTAGTCAAGCCTTGTCCAATC | CTATTGACATTGGTTCTCCTGGTA |
|  | g5037.t2 & g5038.t2 set 9 | AATGGGAACTTGACGCTGG | TGGTGGTGGTTTTGCTGG |
|  | g5037.t2 & g5038.t2 set 10 | GCAGATACATCAACAACATTTACATC | ACACCTTTTCCATCCGTATTCC |
|  | g5037.t2 & g5038.t2 set 11 | GTACCCACCTCAACAACCTC | TTGGCTCAATGGACTCTTGG |
| **M13** | | GTAAAACGACGGCCAGT | CAGGAAACAGCTATGAC |

**Table S2**. Sequences of primers designed for RT-qPCR analysis using the obtained RWA Sanger sequences from single *D. noxia* genes of each *DNMT* subfamily and TET as template. Reference gene sequences and the literature citing them are also noted.

| **Gene target** | ***D. noxia* gene** | **Forward (F) primer sequence (5’-3’)** | **Reverse (R) primer sequence (5’-3’)** | **T_a_**  **(°C)** | **Reference** |
| --- | --- | --- | --- | --- | --- |
| ***DNMT* subfamily** |  |  |  |  |  |
| 1 | g3935.t2 | AGATGTATTACACGTATGGGC | AGACGTTTATAGGCTCAGGA | 56.8 | Current study |
| 2 | g9354.t2 | GCTCTGAGTCAGTCGGGTTT | CACACGGCTGCACAATTTAT | 58.3 | Current study |
| 3A | g20164.t1 | GGCTTTTGAAACAAGTGCTGC | AACCGGCTTCTTTGTTGGAC | 58.3 | Current study |
| *TET* | g5037.t2 | GGCACCCAAAGTACATCCGA | GCGTGTAGTTCCTGCTTTGC | 57.0 | Current study |
| *L27* | g7580.t2 | ACCAGCACGATTTTACCAGATTTC | CGTAGCCTGCCCTCGTGTA | 56.3 | Sinha and Smith 2014 |
| *L32* | g3998.t2 | CGTCTTCGGACTCTGTTGTCAA | CAAAGTGATCGTTATGACAAACTCAA | 56.3 | Shakesby *et al*. 2009 |

**Table S3.** Results from whole genome bisulfite sequencing (*n* = 3). Indicated is the total number of sequenced cytosine bases, the number of bases and percentage of cytosine bases that are methylated.

| **Biotype** | **SA1** | **SA1** | **SA1** | **TOTAL** | **SAM** | **SAM** | **SAM** | **TOTAL** |
| --- | --- | --- | --- | --- | --- | --- | --- | --- |
| **Total** | 3,404,633,702 | 1,744,113,509 | 1,697,849,872 | **6,846,597,083** | 1,743,253,376 | 1,706,831,793 | 3,929,880,530 | **7,379,965,699** |
| **Methylated** | 25,728,030 | 22,901,532 | 22,231,900 | **70,861,462** | 21,732,821 | 22,244,539 | 30,096,579 | **74,073,939** |
| **Methylation (%)** | 0.755677 | 1.31308 | 1.30941 |  | 1.24668 | 1.30326 | 0.76584 |  |

**Table S4.** Results from whole genome bisulfite sequencing (*n* = 3). Indicated is the average number of bases and the number and percentage of cytosine bases that are methylated.

| **Parameter** | **RWA biotype** | | |
| --- | --- | --- | --- |
|  | **SA1** | **SAM** |  |
| **Total bases** | 2,282,199,028 ±  97,232,134 | 2,459,988,566 ± 1,273,094,035 |  |
| **Methylated bases** | 23,620,487 ±1,855,641 | 24,691,313 ± 4,688,084 |  |
| **Percentage of methylation** | 1.126 ± 0.321 % | 1.105 ± 0.295% |  |

**Table S5.** Results from whole genome bisulfite sequencing (*n* = 3). Indicated is the total number of sequenced cytosine bases, and the number and percentage of bases that are methylated in each of the different methylation contexts (i.e., CpG, CHG and CHH).

|  | **RWA biotype** | | | | | |
| --- | --- | --- | --- | --- | --- | --- |
|  | **SA1** | | | **SAM** | | |
| **Context of methylation** | **CpG** | **CHG** | **CHH** | **CpG** | **CHG** | **CHH** |
| **Total bases** | 453,917,795 ± 366,873,653 | 330,615,912 ± 209,356,753 | 1,497,665,321 ± 396,137,039 | 501,515,675 ± 439318412 | 360,963,386 ± 259,939,966 | 1,597,509,505 ± 573,836,120 |
| **Methylated bases** | 18,188,159.33 ±3,856,496 | 740,468.66  ± 25,881 | 4,691,859 ± 2,042,671 | 19,298,939.33 ± 6,070,887 | 776,601.67 ± 149,872 | 4,615,772 ± 1,548,876 |
| **Percentage of methylation** | 5.257 ± 2.319 % | 0.275 ± 0.122 % | 0.348 ± 0.198 % | 5.119 ± 2.184% | 0.266 ± 0.106% | 0.332 ± 0.179% |

**Table S6.** Results from whole genome bisulfite sequencing (*n* = 3). Indicated is the total number of sequenced cytosine bases, and the number and percentage of cytosine bases that are methylated in genic and intergenic regions.

|  | **RWA biotype** | | | |
| --- | --- | --- | --- | --- |
|  | **SA1** | | **SAM** | |
| **Region of methylation** | **Genic** | **Intergenic** | **Genic** | **Intergenic** |
| **Total bases** | 931,707,288 ± 504,748,343 | 1,350,491,740 ± 467,733,575 | 1,015,675,933 ± 639,856,956 | 1,444,312,634 ± 633,310,211 |
| **Methylated bases** | 13,591,802 ± 3,433,743 | 10,028,685 ± 1,606,244 | 14,499,028 ± 5,262,393 | 10,192,285 ± 609,023 |
| **Percentage of methylated bases** | 1.595 ± 0.379% | 0.820 ± 0.336% | 1.573 ± 0.347% | 0.797 ± 0.313% |

**Table S7.** Results from whole genome bisulfite sequencing (*n* = 3). Indicated is the total number of sequenced cytosine bases, and the number and percentage of cytosines that are methylated in both the top and bottom strands.

|  | **RWA biotype** | | | |
| --- | --- | --- | --- | --- |
|  | **SA1** | | **SAM** | |
| **Methylated strand** | **Top** | **Bottom** | **Top** | **Bottom** |
| **Total bases** | 1,129,560,026 ± 480,651,190 | 1,152,639,002 ± 491,683,499 | 1,218,217,681 ± 631,346,828 | 1,241,770,885 ± 641,748,388 |
| **Methylated bases** | 11,818,572.33 ± 861,981 | 11,801,915 ± 993,856 | 12,370,953 ± 2,312,637 | 12,320,359.33 ± 2,375,455 |
| **Percentage of methylated bases** | 1.139 ± 0.329% | 1.113 ± 0.313% | 1.119 ±0.302 | 1.091 ± 0.288% |

**Table S8.** Results from whole genome bisulfite sequencing (*n* = 3). Indicated is the total number of sequenced cytosine bases, and the number and percentage of cytosine bases that are methylated in genic regions (i.e., either exonic or intronic).

|  | **RWA biotype** | | | |
| --- | --- | --- | --- | --- |
|  | **SA1** | | **SAM** | |
| **Genic region methylated** | **Exon** | **Intron** | **Exon** | **Intron** |
| **Total bases** | 421,831,158 ± 356,122,277 | 250989142 ± 105575120 | 467,652,038 ± 431,095,157 | 271,275,455.3 ± 142,455,559 |
| **Methylated bases** | 7,500,021.667 ± 3,373,530 | 2741161 ± 105799 | 8,153,134 ± 4,472,927 | 2,834,042 ± 222,933 |
| **Percentage methylation** | 2.168 ± 0.694% | 1.217 ± 0.444% | 2.158 ± 0.674% | 1.191 ± 0.419% |

**Table S9.** Results from whole genome bisulfite sequencing (*n* = 3). Indicated is the number of genes that methylated in one biotype but not the other, as well as the average methylation percentage of those genes. Also indicated is the context of the methylation (i.e., CpG, CHG or CHH).

| **Parameter** | **RWA biotype** | | | | | |
| --- | --- | --- | --- | --- | --- | --- |
|  | **SA1** | | | **SAM** | | |
| **Context of methylation** | **CpG** | **CHG** | **CHH** | **CpG** | **CHG** | **CHH** |
| **Uniquely methylated genes** | 2,289.67 ± 444.82 | 3,227.00 ± 305.96 | 1,244.67 ± 259.02 | 2,205.67 ± 381.00 | 3,160.33 ± 322.32 | 1,226.33 ± 414.57 |
| **Percentage level of methylation per context** | 0.538 ± 0.121 | 0.457 ± 0.026 | 0.451 ± 0.053 | 0.473 ± 0.043 | 0.459 ± 0.036 | 0.440 ± 0.048 |
